# Optogenetics reveals a role for accumbal medium spiny neurons expressing dopamine D2 receptors in cocaine-induced behavioral sensitization

**DOI:** 10.3389/fnbeh.2014.00336

**Published:** 2014-10-13

**Authors:** Shelly Sooyun Song, Byeong Jun Kang, Lei Wen, Hyo Jin Lee, Hye-ri Sim, Tae Hyong Kim, Sehyoun Yoon, Bong-June Yoon, George J. Augustine, Ja-Hyun Baik

**Affiliations:** ^1^Department of Life Sciences, Molecular Neurobiology Laboratory, School of Life Sciences and BiotechnologyKorea University, Seoul, South Korea; ^2^Center for Functional Connectomics (CFC)KIST, Seoul, South Korea; ^3^Lee Kong Chian School of Medicine, Nanyang Technological UniversitySingapore, Singapore; ^4^Institute of Molecular and Cell BiologySingapore, Singapore

**Keywords:** optogenetics, medium spiny neurons, dopamine D2 receptors, cocaine, drug addiction

## Abstract

Long-lasting, drug-induced adaptations within the nucleus accumbens (NAc) have been proposed to contribute to drug-mediated addictive behaviors. Here we have used an optogenetic approach to examine the role of NAc medium spiny neurons (MSNs) expressing dopamine D2 receptors (D2Rs) in cocaine-induced behavioral sensitization. Adeno-associated viral vectors encoding channelrhodopsin-2 (ChR2) were delivered into the NAc of D2R-Cre transgenic mice. This allowed us to selectively photostimulate D2R-MSNs in NAc. D2R-MSNs form local inhibitory circuits, because photostimulation of D2R-MSN evoked inhibitory postsynaptic currents (IPSCs) in neighboring MSNs. Photostimulation of NAc D2R-MSN *in vivo* affected neither the initiation nor the expression of cocaine-induced behavioral sensitization. However, photostimulation during the drug withdrawal period attenuated expression of cocaine-induced behavioral sensitization. These results show that D2R-MSNs of NAc play a key role in withdrawal-induced plasticity and may contribute to relapse after cessation of drug abuse.

## Introduction

Dopamine (DA) signaling is associated with reward expectation and goal-directed behavior (Wise, 2004; Goto and Grace, 2005; Berridge, 2007). One of the well-known pathologies of dopaminergic disorders is drug addiction (Robinson and Berridge, 1993, 2003). Following repeated exposure to addictive substances, adaptive changes occur at the molecular and cellular level in the DA mesolimbic pathway; these can lead to drug dependence, which is a chronic, relapsing disorder in which compulsive drug-seeking and drug-taking behaviors persist despite their serious negative consequences (Thomas et al., 2008; Baik, 2013). Characterization of the modifications that take place in the mesolimbic dopaminergic system is thus key to understanding drug addiction.

Dopamine D1 receptors (D1R) and D2 receptors (D2R) are highly expressed in the medium spiny neurons (MSNs) of the striatum. It has been suggested that long-lasting drug-induced adaptations in the ventral striatum, better known as the nucleus accumbens (NAc), contribute to the development of addiction as well as drug-seeking and relapse behaviors (Lobo and Nestler, 2011; Smith et al., 2013). Dopaminergic cell bodies from the ventral tegmental area mostly innervate the NAc. Over 95% of the cells within the NAc are MSNs, which receive excitatory inputs from four major brain regions: the prefrontal cortex, the ventral subiculum of the hippocampus, the basolateral amygdala, and the thalamus (Sesack and Grace, 2010; Lüscher and Malenka, 2011). MSNs within the NAc can be divided into two major subpopulations: direct pathway MSNs that express D1Rs and project directly to midbrain DA areas, and indirect pathway MSNs that express D2Rs and project to the ventral pallidum (Kreitzer and Malenka, 2008; Sesack and Grace, 2010; Lüscher and Malenka, 2011; Smith et al., 2013). Because MSN are GABAergic, activation of MSNs neurons will inhibit their downstream targets which are also GABAergic (Chevalier and Deniau, 1990). Therefore, activation of D1R-MSNs will excite midbrain DA neurons, which then contributes to the regulation of reward-related behaviors (Lüscher and Malenka, 2011; Bocklisch et al., 2013).

Recent studies using genetically engineered mice that express Cre recombinase in a cell-type specific manner have revealed different roles for D1R-MSNs and D2R-MSNs in cocaine addiction behaviors. Such mice enable genetic targeting of specific toxins, optogenetic probes or DREADD (designer receptors exclusively activated by a designer drug) to selectively manipulate D1R-MSN or D2R-MSN. This approach has led to some consensus about the role of MSNs in addictive behaviors: D1R-MSNs apparently promote addictive behaviors, while no specific role (or an inhibitory role) in the development of drug-induced addictive behaviors has been suggested for D2R-MSNs (Hikida et al., 2010; Lobo et al., 2010; Ferguson et al., 2011; Bock et al., 2013). Cocaine exposure apparently induces synaptic modification and alterations in gene expression in both MSN populations (Lobo et al., 2010; Lobo and Nestler, 2011; Grueter et al., 2013). Although it appears that D1R-MSNs and D2R-MSNs play opposing roles in cocaine-mediated addictive behaviors, the precise role of D2R-MSNs is not clear.

Previously it has been shown that D2R knockout (KO) mice display normal cocaine-mediated behavioral sensitization and cocaine-seeking behaviors, with only a slight decrease in sensitivity caused by the absence of D2R (Baik et al., 1995; Chausmer et al., 2002; Sim et al., 2013). However, exposure to stress during drug withdrawal suppresses expression of cocaine-induced behavioral sensitization as well as cocaine-seeking and relapse behaviors in D2R KO mice (Sim et al., 2013). Specific knock-down of D2R in the NAc does not affect basal locomotor activity, nor cocaine-induced behavioral sensitization, but does confer the ability of stress to inhibit expression of cocaine-induced behavioral sensitization (Sim et al., 2013). These findings strongly suggest that blockade of D2R in the NAc does not prevent cocaine-mediated behavioral sensitization. Rather, it appears that D2R in the NAc play a distinct role in regulation of the stress-triggered synaptic modifications during withdrawal that lead to an increase in cocaine-seeking and relapse behaviors (Sim et al., 2013).

Here we have used optogenetics to further evaluate the role of NAc D2R-MSNs in cocaine-induced behavioral sensitization. Using brain slices, we find that photostimulation of D2R-MSNs activates local inhibitory circuits within NAc involving neighboring MSNs. Photostimulation of NAc D2R-MSNs *in vivo* affects neither the initiation nor the expression of cocaine-induced behavioral sensitization. However, repetitive activation of NAc D2R-MSNs during the drug withdrawal period attenuates cocaine-induced addictive behavior. Our results show that D2R-MSNs of NAc play a key role in withdrawal-induced plasticity and may contribute to relapse after cessation of drug abuse.

## Materials and methods

### Mice

D2-Cre BAC transgenic mice on a C57Bl/6 background were obtained from MMRRC (Mutant Mouse Regional Resource Centers, B6.FVB(Cg)-Tg(Drd2-cre)ER44Gsat/Mmucd). In behavioral experiments, littermates lacking the D2-Cre transgene were used as controls for the D2-Cre mice. Mice were maintained in a specific pathogen-free barrier facility under constant conditions of temperature and humidity, and on a 12-h light, 12-h dark schedule. Animal care and handling were performed in accordance with standards approved by the Institutional Animal Care and Use Committees of Korea University and KIST.

### Virus vector preparation

pAAV-EF1a-DIO-hChR2(H134R)-EYFP-WPRE was generously provided by Karl Deisseroth (Stanford Univ.). For preparation of AAV, HEK293T cells were grown in DMEM media with antibiotics and FBS. The day before transfection, four plates beyond 90% confluence from 10-cm dishes were plated onto five 15-cm dishes and incubated for 18–22 h or until 60 to 70% confluence. HEK293T cells were transfected with pAAV-DIO-ChR2-EYFP, pAAV-DJ and pHelper using jetPEI transfection reagent (QBiogene). The DNA/DMEM/PEI cocktail was vortexed and incubated at room temperature for 20 min. After incubation, the transfection mixture was added to each 15 cm dish. Transfected cells were harvested 48 h after transfection and incubated with 0.5% sodium deoxycholate (Sigma; D6750) and 50 units/ml of benzonase nuclease (Sigma; E1014) at 37°C for 1 h. After removing cellular debris by centrifuging at 3000 × g for 15 min, the supernatant was filtered through a 0.45 mm PVDF filter (Millipore). Purification of AAV- DJ particles was performed using HiTrap heparin affinity columns (GE Healthcare). For concentration of AAV, Amicon ultra-15 centrifugal filter units with a 100,000 molecular weight cutoff were used. Concentrated virus aliquoted and frozen for storage at −80°C. The final viral concentrations was 3~6 × 10^12^ virus particles per ml for each AAV.

### Stereotaxic injection and optical fiber placement

Animals were anesthetized by i.p. injections of 1.6 µl of Zoletil and 0.05 µl of xylazine (Rompun, Bayer) per gram of body weight and placed in a stereotaxic apparatus (David Kopf Instruments, Tujunga, CA). For injection of viruses, a 31-gauge syringe needle was used to bilaterally infuse 2 µl of virus into NAc at an angle of 0° (AP +1.7; ML ±1.3; DV −4.5) at a rate of 0.1 ul/min. The needle was left in place for 10 min after injection before being slowly withdrawn. The fiber-optic cannula for implantation consisted of a zirconia ferrule (1.25 mm in diameter and 4.5 mm long) and flat tip of an optical fiber (200 µm in diameter). The implantation of the fiber-optic cannula into NAc for illumination of D2-MSNs was performed immediately after injection of viruses. The coordinates for implantation of the fiber-optic cannula were an angle of 0° (AP +1.7; ML ±1.35; DV −4.2) for targeting NAc. To help anchor the optical fiber, two screws were anchored into the skull to the rear of the implantation site of the fiber-optic cannula. To fix the fiber-optic cannula on the skull, C&B Superbond (Sun Medical) was applied to the surface of the skull around the base of the cannula. Once the C&B Superbond hardened, the cannula was released from the holder and dental cement (Poly-F, Dentsply) was applied around the cannula and screws. To close the incision around the cannulation site, Vetbond tissue adhesive (3 M, 7003449) was used. After implantation, mice were given subcutaneous injection of antibiotics (Enrofloxacin, 5 mg/kg, q 12 h) and analgesia (Carprofen, 5 mg/kg, q 24 h) for 3 consecutive days.

### *In vivo* photostimulation

A 200 µm patch cord was connected to the external portion of the chronically implantable optical fiber using a sleeve. Optical fibers were attached through an FC/PC adaptor to a blue laser diode (473 nm, MBL-III 473-150 mW), and light pulses were generated through a stimulator (BNC 575). For photostimulation of ChR2-expressing neurons, the stimulation paradigm was 20 Hz frequency, 5 ms pulse duration and 2–5 mW of light power. Light power emitted from the patch cord was measured using a power meter (PM100D) with a S121C light sensor.

### Behavioral Analysis

Behavioral experiments were performed with male D2-Cre mice at 11–13 weeks of age, with the exception of mice subjected to electrophysiological analysis which were 5–6 weeks of age. Age-matched D2-Cre and Cre negative control mice were injected with virus and housed individually and allowed to acclimate to the cage until the behavioral test. For each manipulation, mice were transferred to the experimental room 60 min before the onset of the experiment to allow for habituation and to reduce stress (brightness of the experimental room was 70 lux). Each experimental apparatus was cleaned with 70% ethanol between experiments to remove any potential odor cues.

### Cocaine sensitization

For initiation of cocaine sensitization, mice were habituated to saline injections (i.p.) for 3 consecutive days and then injected with saline or cocaine (15 mg kg^−1^, i.p) for 5 consecutive days. Mice were injected intraperitoneally (i.p.) with either cocaine hydrochloride (Johnson Mattney, Edinburgh, UK) dissolved in saline (0.9% NaCl) or saline with a 30 G needle. Immediately after each injection, mice were tested for horizontal locomotor activity in an open-field chamber for 30 min. For measurement of the effect of photostimulation on the initiation and expression of sensitization (Figure 5), mice were given blue light illumination bilaterally through dual fiber-optic patch cords onto the NAc for four 3-min periods during 30 min sessions in home cages. Patch cords from the fiber-optic cannula located on the mouse skull was removed and mice were given at least 10 min rest. Mice were then injected with either cocaine or saline (coc 1d-coc 5d). After initiation of sensitization, cocaine was withdrawn for 14 days without any injection of saline. During this withdrawal period, no photostimulation was applied. The expression of behavioral sensitization to cocaine was then determined by injection of a challenge dose of the drug (10 mg kg^−1^, i.p.) after photostimulation of the NAc as illustrated in Figure 5A. To measure the effect of photostimulation during the cocaine withdrawal period (Figure 6), mice were subjected to the same protocol for sensitization as described above (for Figure 5) except photostimulation was given. After initiation of cocaine sensitization, photostimulation was applied to the NAc daily for 1 h during the total withdrawal period of 14 days. After 14 days of withdrawal, all groups of mice were injected with the challenge dosage of cocaine, (10 mg kg^−1^).

**Figure 1:**
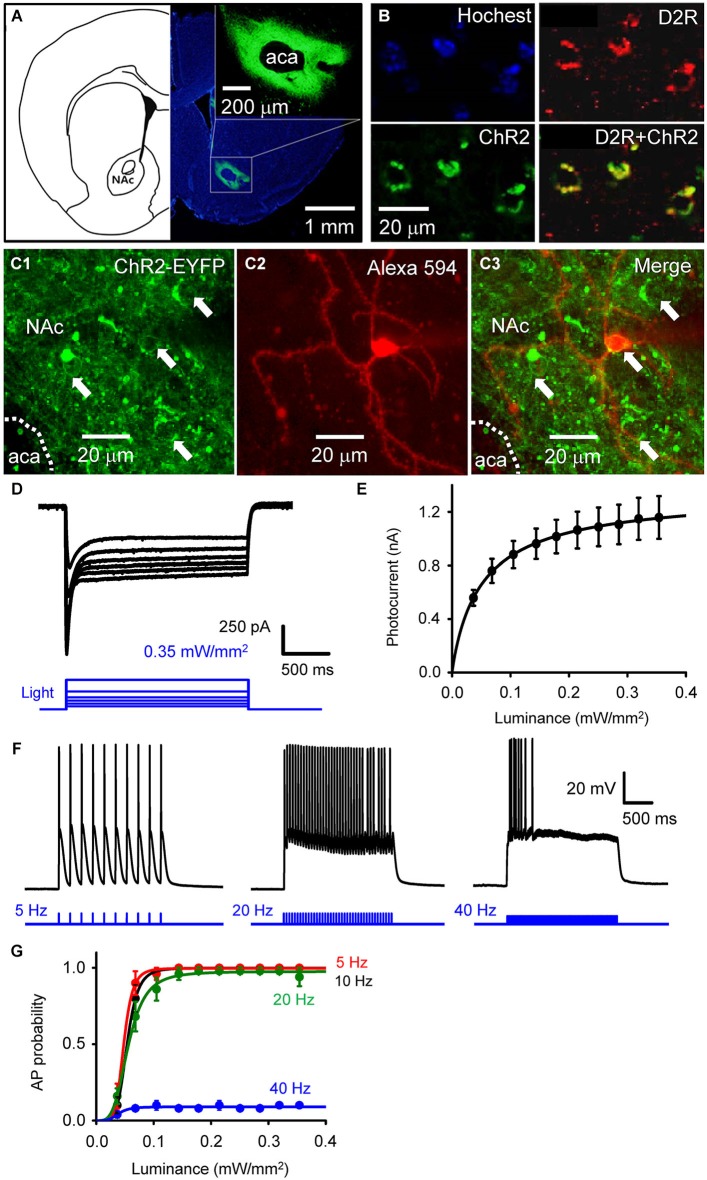
**Selective photostimulation of medium spiny neurons in nucleus accumbens. (A)** Selective expression of ChR2 in NAc D2R neurons by delivery of AAV-DIO-ChR2-EYFP viral vectors. scale bars: background figure, 1 mm: insert, 200 µm. **(B)** Confocal images of D2R-Cre mouse brain slices infected with AAV-DIO-ChR2-EYFP virus showing the co-expression of ChR2 (green) in NAc neurons with dopamine D2 receptors (red) (D2R+ChR2). **(C)** Confocal image of neurons in a living NAc slice. Representative neurons expressing ChR2-EYFP (green) are indicated by arrows (C1 and C3), with one of the cells injected with Alexa594 dye (red) during the course of whole-cell patch recording to identify the neuron’s structure (C2). aca: anterior part of anterior commissure; NAc: nucleus accumbens core. **(D)** ChR2-mediated photocurrent (black traces) evoked by light flashes (470 nm, blue traces at bottom) of various intensities. **(E)** Relationship between peak amplitude of photocurrents and light intensity. Points indicate mean ± S.E.M (*n* = 4), while the curve is fitted with the Hill equation. **(F)** Representative membrane potential responses to trains of light flashes (10 ms, 0.35 mW/mm^2^) at the indicated frequencies. **(G)** Relationship between light intensity and probability of light-evoked action potentials (APs) during trains of light flashes, as in F (*n* = 5). Curves are fitted with the Hill equation.

**Figure 2:**
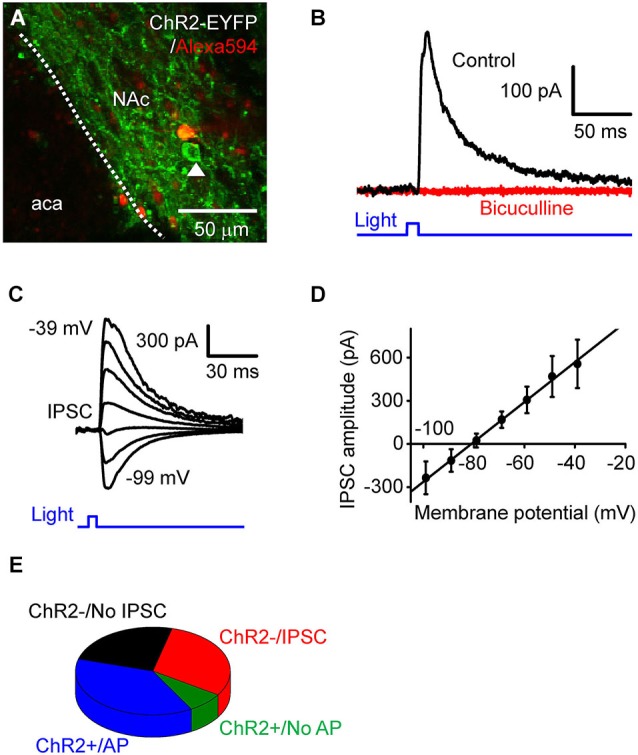
**Photostimulation of D2RCre-MSNs drives local inhibitory circuits. (A)** Confocal image of a live NAc slice, showing a dye-filled neuron that does not express ChR2 and a neighboring cell (arrowhead) that expressed ChR2 and could be photostimulated. **(B)** IPSC (black trace) induced by a light pulse (10 ms duration, 0.35 mW/mm^2^) at a holding potential of −60 mV. The IPSC was blocked by bath application of bicuculline (red trace). **(C)** IPSC evoked by light pulses (10 ms, 0.35 mW/mm^2^) when the membrane potential was clamped at 10 mV increments from −99 mV to −39 mV. **(D)** Voltage dependence of light-induced IPSCs. The relationship between IPSC amplitude and membrane potentials (*n* = 6) could be fit by a straight line and had an estimated reversal potential of −81 mV. **(E)** Four different classes of responses of NAc neurons tophotostimulation.

**Figure 3:**
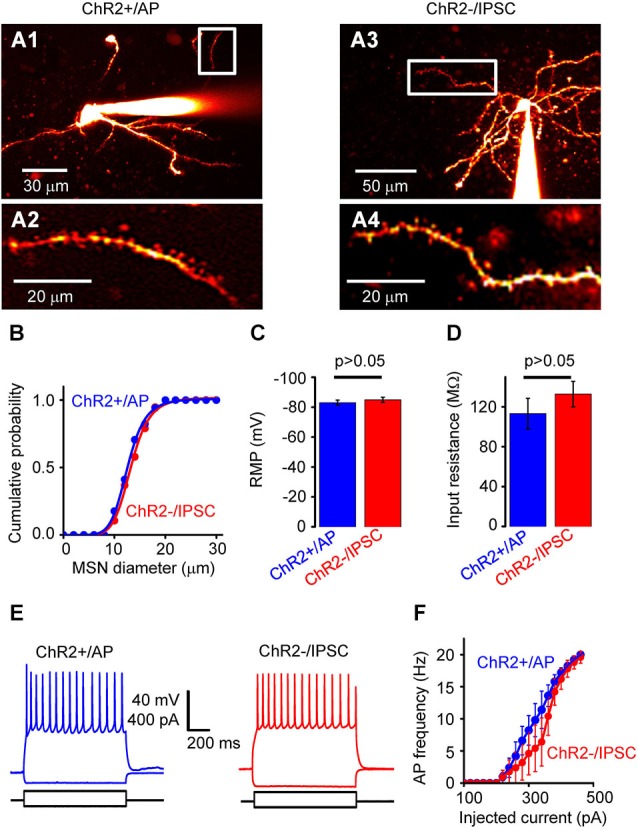
**Properties of NAc cells. (A)** Two-photon fluorescence image of neurons filled with Alexa 594. **(A1)** shows a neuron from the ChR2+/AP group, while **(A3)** shows a neuron from the ChR2−/IPSC group. **(A2)** and **(A4)** are high-magnification images from the regions indicated by white rectangles, showing spiny dendrites of both neuron types. **(B)** Distribution of somatic diameter of MSNs in ChR2+/AP (blue) and ChR2−/IPSC (red) groups. There was no significant difference between two groups. **(C)** Resting membrane potential (RMP) of neurons from ChR2+/AP (blue) and ChR2−/IPSC (red) groups (*n* = 10 for each group). There was no significant difference in values between the two groups. **(D)** Membrane input resistance of neurons from ChR2+/AP (blue) and ChR2−/IPSC (red) groups (*n* = 6 for each group). There was no significant difference in values between the two groups. **(E)** Representative examples of membrane responses to current pulses recorded from ChR2+/AP (blue) and ChR2−/IPSC (red) cells. **(F)** Relationship between stimulating current intensity and action potential (AP) firing frequency for cells in group of ChR2+/AP (blue) and ChR2−/IPSC (red; *n* = 6 for each group). There was no significant difference between the two groups of cells.

**Figure 4:**
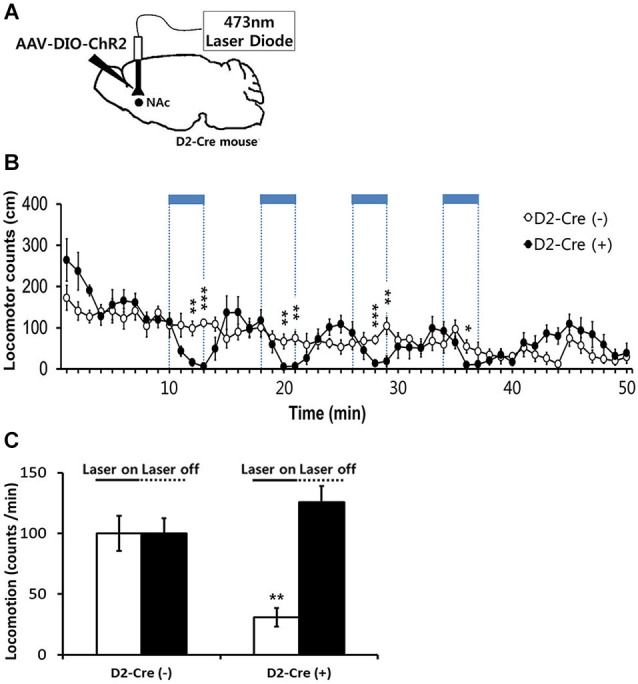
**Effects of in-vivo optogenetic activation of D2-MSNs in NAc on basal locomotor activity**. **(A)** Sagittal view of D2 Cre mice injected at the NAc with AAV-DIO-ChR2-EYFP followed by bilateral implantation of fiber optic cannula. 473 nm blue light stimulation (2 mW, 5 ms, 20 Hz) was delivered via dual fiber-optic patch cords attached to the implanted cannula. **(B)** Locomotor counts during blue-light on/off periods in D2-Cre positive D2-Cre(+) and D2-Cre negative D2-Cre(−) mice (*n* = 6 mice per group). Blue-light pulses (2 mW, 5 ms, 20 Hz) were delivered for four 3-min periods during the 50-min session in the locomotor activity chamber as indicated by blue bars. **(C)** Locomotor counts per minute during blue-light illumination (four 3-min epochs, 2 mW, 5 ms, 20 Hz) and during the laser off period for D2 Cre(+) and D2 Cre(−) mice. Two-tailed Student’s *t*-test for c and d, * *P* < 0.05, ** *P* < 0.01, *** *P* < 0.001 vs. D2-Cre(−) group. Data are means ± s.e.m.

**Figure 5:**
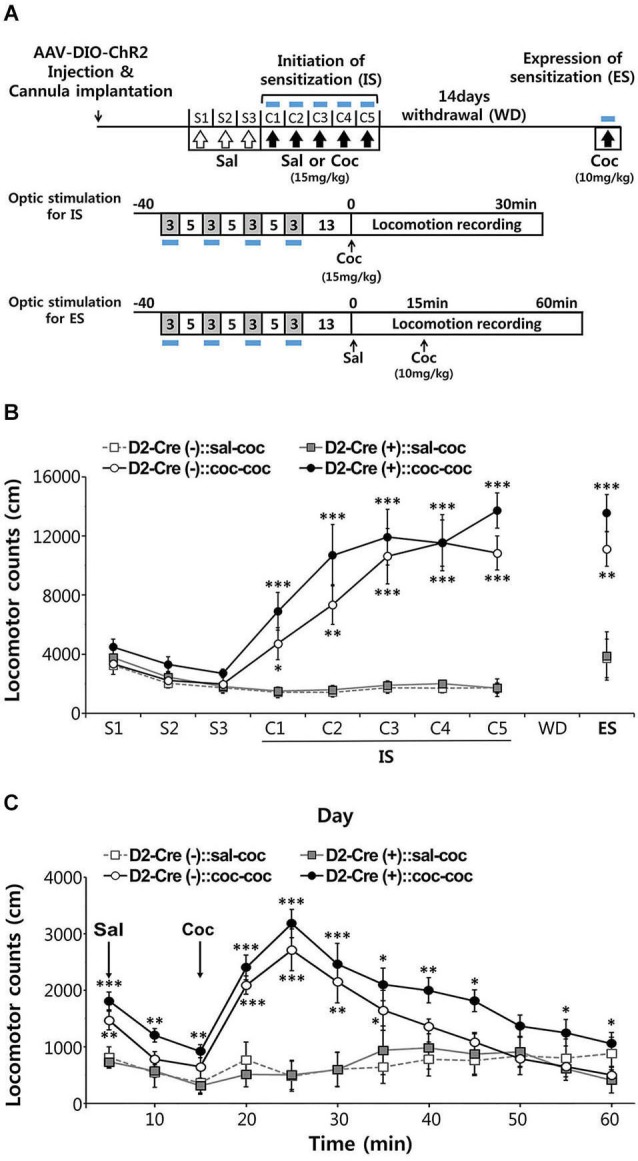
**Effects of activation of D2-MSN during sensitization to cocaine. (A)** Experimental scheme for photo-stimulation of D2-MSNs during initiation and expression of sensitization to cocaine. Blue-light illumination (2~5 mW, 5 ms, 20 Hz) was delivered for four 3-min periods during 40-min sessions before measuring locomotion as indicated by blue bars. Sal, saline; Coc, cocaine; IS, initation of sensitization; WD, withdrawal; ES, expression of sensitization. **(B)** The effects of photo-stimulation during the initiation of behavioral sensitization (IS) with five daily injections of either saline or cocaine (15 mgkg^−1^, i.p.) and during the expression of behavioral sensitization (ES) induced by challenge dose of cocaine (10 mgkg^−1^, i.p.) after 14 days of drug withdrawal (For coc 1d-5d: cocaine effect *F*_(1,11)_ > 24.55, *p* < 0.05. For ES: cocaine effect in *F*_(1,11)_ = 40.14, *p* < 0.0001; cocaine × D2-Cre interaction *F*_(1,11)_ = 0.7147, *p* = 0.407, *n* = 6~7). Two-way analysis of variance (ANOVA) *post hoc* test: * *P* < 0.05, ** *P* < 0.01, *** *P* < 0.001 vs. saline-treated group). Data are means ± s.e.m. **(C)** Detailed time course of locomotor activity for D2-Cre(–) or D2-Cre(+) mice during ES to cocaine (10 mgkg–1, i.p.) following blue-light illumination. Arrows denote time of injection with either saline or cocaine (For 20–50 m: cocaine effect *F*_(1,11)_ > 1.812, *P* < 0.1919, *n* = 6~7). Two-way analysis of variance (ANOVA) *post hoc* test: * *P* < 0.05, ** *P* < 0.01, *** *P* < 0.001 vs. saline-treated group). Data are means ± s.e.m.

**Figure 6:**
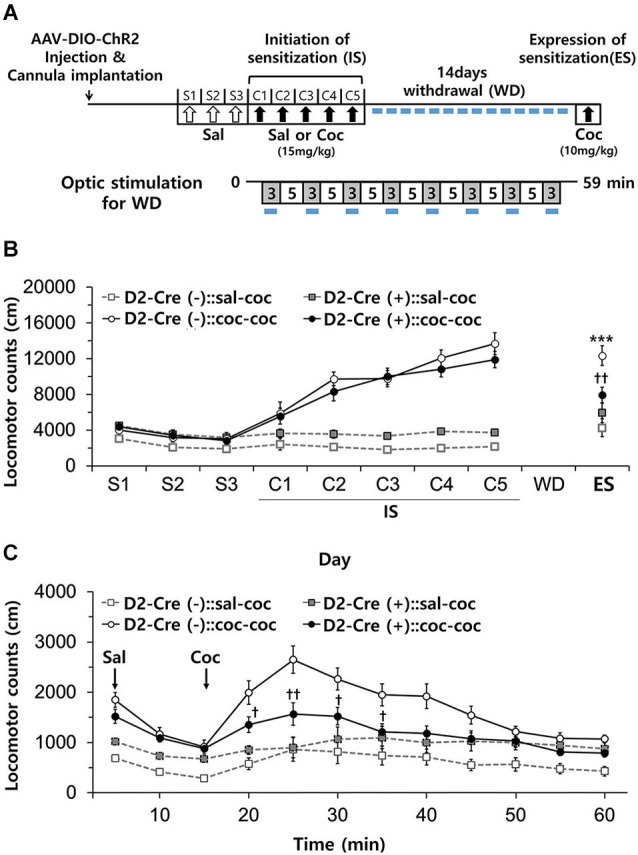
**Effects of activation of D2-MSN during withdrawal to repeated cocaine exposure. (A)** Experimental scheme for photo-stimulation of D2-MSNs during withdrawal to cocaine. Blue-light illumination (2~5 mW, 5 ms, 20 Hz) was delivered for eight 3-min periods (indicated by blue bars) during 59 min sessions before measuring locomotion. Sal, saline; Coc, cocaine; IS, initation of sensitization; WD, withdrawal; ES, expression of sensitization. **(B)** Effect of daily photo-stimulation of D2-MSNs during the withdrawal period on the expression of sensitization (ES) to cocaine. Locomotor activity of D2-Cre(+) or D2-Cre(−) mice (*n* = 8–11 per group) was measured during the initiation and expression of behavioral sensitization to cocaine as in **Panel B** (For ES: cocaine effect *F*_(1,35)_ = 24.34, *p* < 0.0001; cocaine × D2-Cre interaction *F*_(1,35)_ = 8.93, *p* = 0.0051). **(C)** Detailed time course of locomotor activity for D2-Cre(−) or D2-Cre(+) mice during ES to cocaine (10 mg/kg, i.p.) (For 15 m–60 m: cocaine × D2-Cre interaction *F*_(1,35)_ > 4.97, *p* < 0.032). Two-way analysis of variance (ANOVA) *post hoc* test: * *P* < 0.05, *** *P* < 0.001 vs. saline-treated group; ^†^ *P* < 0.05, ^††^ *P* < 0.01 vs. D2-Cre(−) group). Two-way ANOVA *post hoc* test: *** *P* < 0.001 vs. saline-treated group; ^†^ *P* < 0.05, ^††^ *P* < 0.01 vs. D2-Cre(−) group. All data are means ± s.e.m.

### Immunofluorescence and confocal laser microscopy

For immunofluorescence, mice were anesthetized with Zoletil (Virbac, 1.6 µl/g, intraperitoneally) and 0.05 µl/g Rompun (Bayer) and perfused with filter-sterilized 0.1 M PBS followed by fixation using 4% paraformaldehyde/PBS solution (Sigma). The brain was then removed and post-fixed for 4 h with ice-cold fixative as above. The brains were then dehydrated in 30% sucrose/0.1 M PBS for 2 days. Brains were then frozen and 40-µm-thick consecutive coronal sections were prepared on a cryostat (Leica CM 1900, Germany). Sections (40 µm) were blocked for 1 h in 0.1 M PBS containing 5% normal goat serum and 0.2% Triton X-100 and incubated with rabbit polyclonal anti-D2R (1:500, Millipore, AB5084P) at 4°C overnight. After washes with PBS containing 0.2% Triton X-100, samples were incubated at RT for 1 h with Alexa Fluor 568 goat anti-rabbit IgG (1:500; Molecular Probes, Eugene, OR, USA) and 0.2 µl/ml 4, 6-diamidino-2-phenyl-indole HCl (DAPI; Sigma, St. Louis, MO, USA) in PBS containing 1% normal goat serum and 0.2% Triton X-100. As a negative control, samples were incubated with DAPI and the secondary antibody only. Sections were examined on a C1 Plan Apo × 40/1.4 water confocal laser scanning system (LSM 700, Zeiss, Berlin, Germany).

### Electrophysiology and photostimulation in nucleus accumbens slices

Mice were used for experiments 4 weeks after virus injection, to achieve optimal expression of ChR2-EYFP. Mice were then anesthetized and decapitated for preparation of acute brain slices. The brain was quickly removed and immediately placed in ice-cold cutting solution containing (in mM) 250 Sucrose, 26 NaHCO_3_, 10 D-Glucose, 3 Myo-inositol, 2.5 KCl, 2 Na-pyruvate, 1.25 NaH_2_PO_4_, 0.5 Ascorbic acid, 1 Kynurenic acid and 7 MgCl_2_ which was bubbled with 95% O_2_/5% CO_2_ (pH = 7.4). Coronal brain slices (250 µm thick) containing the NAc were prepared using a vibratome (Leica VT 1200 S) and were then incubated in gassed artificial cerebrospinal fluid (ACSF) containing (in mM): 11 D-glucose, 125 NaCl, 25 NaHCO_3_, 1.25 NaH_2_PO_4_, 2.5 KCl, 1.25 MgCl_2_ and 2.5 CaCl_2_ at 34°C for 1 h before recording. Slices were then transferred to a submersion recording chamber in which O_2_-saturated ACSF solution was continuously superfused. Cells in NAc and VTA were visualized using a 2-photon microscope (Olympus FV1000 MPE, Tokyo, Japan) equipped with a 25X water immersion lens and infrared DIC optics. Whole-cell patch clamp recordings were obtained from NAc cells with a Multiclamp 700B amplifier and Digidata 1440A digitizer (Molecular Devices, LLC). Data were sampled using pCLAMP 10.2 software and further analyzed using Clampfit 10.2 software (Molecular Devices, LLC). Patch electrodes with resistances between 3–5 MΩ were filled with an internal solution containing (in mM): 130 K-gluconate, 2 NaCl, 2 MgCl_2_, 20 HEPES, 4 Na_2_ATP, 0.4 Na_3_GTP, 0.5 EGTA and 10 Na_2_- phosphocreatine, with pH adjusted to 7.3 using 1 N KOH. Bicuculline (10 µM) was bath-applied to brain slice to block GABA receptors in a subset of experiments.

NAc cells expressing ChR2-EYFP were photostimulated by a LED light source (460 ± 27 nm, UHP-Mic-LED-460, Prizmatix). Blue light from the LED was further filtered and attenuated by a filter cube equipped with an excitation filter (470–495 nm); flashes of light (10 ms duration, 0.0366–0.354 mW/mm^2^) were delivered to the brain slice via the 25X objective lens at frequencies of 5–40 Hz. In a subset of experiments, photocurrents were measured in ChR2-expressing cells in response to 2 s duration light flashes.

### Statistical Analysis

Data are presented as means ± s.e.m. and were analyzed with the two-tailed Student’s *t*-test, or with two-way analysis of variance followed by Bonferroni’s *post hoc* test. A *P*-value of <0.05 was considered statistically significant.

## Results

### Selective photostimulation of medium spiny neurons in nucleus accumbens

To determine the role of NAc D2R-MSNs in cocaine-mediated addictive behaviors, we used an optogenetic approach to stimulate NAc D2R neurons. To selectively control the activity of D2R-MSNs in NAc by light, viral vectors coding AAV-DIO-ChR2-EYFP were stereotaxically injected into the NAc of D2R-Cre BAC transgenic mice. 4 weeks after viral injection, robust expression of ChR2-EYFP was observed in the NAc (Figure 1A). The specificity of ChR2 expression in D2R-MSNs was confirmed by immunofluorescence confocal analysis: expression of YFP-tagged ChR2 was co-localized with D2R in NAc (Figure 1B), showing that ChR2 was expressed in D2R-expressing neurons in NAc.

Although such an approach has been used in other studies (e.g., Lobo et al., 2010), the details of virus injection procedures will vary from one lab to another, making it important to document optogenetic control under our specific experimental conditions. We assessed the functional expression of ChR2 by making whole-cell patch clamp recordings from MSNs in NAc slices. MSNs were identified by: (1) a relatively hyperpolarized resting membrane potential (RMP), typically more negative than −80 mV; (2) a regular pattern of AP firing in response to applied current pulses; (3) long latency to firing of the first AP during a current pulse; (4) absence of a voltage “sag” during hyperpolarization caused by a hyperpolarization-activated cation current (I_h_); and (5) relatively small size of their cell bodies (Chang and Kitai, 1985; O’Donnell and Grace, 1993; Le Moine and Bloch, 1996; Taverna et al., 2008). Blue light (470 nm) was applied over the entire field of view (0.78 mm^2^) while voltage-clamping the MSNs at a holding potential of −69 mV. Some MSNs expressed ChR2, evident as YFP fluorescence in their somata (arrows in Figures 1C1,C3). Such neurons exhibited substantial photocurrents, with brighter light stimuli eliciting larger photocurrents (Figure 1D). The relationship between peak photocurrent amplitude and light intensity (Figure 1E) had a half-maximal light sensitivity of 0.054 ± 0.0023 mW/mm^2^ and a maximal peak amplitude of 1.16 ± 0.16 nA (mean ± s.e.m., *n* = 4).

Under current-clamp conditions, MSNs expressing ChR2 fired APs reliably in response to trains of light pulses (10 ms duration; Figure 1F). Under these conditions, light intensities greater than 0.1 mW/mm^2^ were sufficient to evoke APs (Figure 1G, *n* = 5). APs were reliably evoked at photostimulation frequencies up to 20 Hz, while at 40 Hz the light-induced responses summed to cause a sustained depolarization that was less effective at evoking APs (Figures 1F,G).

### Photostimulation of D2R-MSNs drives local inhibitory circuits

To investigate the consequences of D2R-MSNs activity on local circuits in NAc, we photostimulated presynaptic MSN expressing ChR2 while measuring postsynaptic responses in ChR2-negative MSNs (Figure 2A). The neuron shown in Figure 2A does not express ChR2, as indicated by the absence of EYFP fluorescence as well as the absence of short-latency photocurrents like those shown in Figure 1D. However, when the postsynaptic MSNs were held as a potential of −69 mV, 10 ms duration light flashes evoked outward currents after a latency of 9.0 ± 0.42 ms (Figure 2B, *n* = 15). To determine the nature of these responses, the postsynaptic membrane potential was varied between −99 mV to −39 mV, while a light flash was applied (Figure 2C). Light-induced responses varied with membrane potential (Figure 2D, *n* = 6) and reversed their polarity at −81 ± 3.4 mV. Given that the equilibrium potential for chloride ions is −80 mV under our ionic conditions, the light-induced outward currents could be due to chloride flux mediated by postsynaptic GABA_A_ receptors. To test this possibility, the GABA_A_ receptor antagonist bicuculline (10 µM) was added to the external solution. This drug completely blocked light-induced responses (Figure 2B), confirming that the light-induced responses were GABAergic inhibitory postsynaptic currents (IPSCs).

Based on their responses to photostimulation, the MSNs that we recorded from could be classified into one of 4 groups: (1) cells expressing a sufficient amount of ChR2 to fire APs in response to photostimulation (ChR2+/AP), which were described above; (2) cells expressing a small amount of ChR2, which evinced a subthreshold depolarization in response to light (ChR2+/No AP); (3) silent cells that had no expression of ChR2 but received light-induced IPSCs from presynaptic MSNs expressing ChR2 (ChR2−/IPSC); and (4) ChR2-negative cells that did not exhibit IPSCs in response to photostimulation of other MSNs (ChR2−/No IPSC). The relative proportion of cells in each of these categories is shown in Figure 2E (*n* = 53). Overall, nearly half of the cells (45.3%) expressed ChR2 (sum of groups (1) and (2)). None of the MSNs that we recorded exhibited both photocurrents and IPSCs in response to photostimulation; this indicates that D2R-positive MSNs do not innervate other members of this same cell population within the NAc.

This classification of responses to light indicates that photostimulation of ChR2+/No AP cells (group 2) and ChR2−/No IPSC cells (group 4) will not generate any electrical signals that could contribute to circuit activity. Thus, to define the effects of photostimulation on circuit function, we characterized in detail the properties of ChR2+/AP MSNs (group 1), which will generate APs when the NAc is photostimulated, and ChR2−/IPSC cells (group 3), which are postsynaptic to the ChR2+/AP MSNs because they receive light-induced IPSCs. ChR2+/AP and ChR2−/IPSC cells in NAc were both identified as spiny neurons (Figure 3A). There were no significant differences in the morphological or electrophysiological properties of neurons in these two groups. For example, the somata of the neurons in those two groups were similar in size (Figure 3B). In addition, their RMPs (−83.0 ± 1.7 vs. −85.0 ± 1.8 mV; mean ± s.e.m; *n* = 10, Figure 3C) and input resistances (113 ± 15 vs. 133 ± 13 MΩ, *n* = 6, Figure 3D) were also not different (*p* > 0.05 two-tailed Student’s *t*-test) while their AP firing patterns in response to current pulses (Figures 3E,F) were also similar (*p* > 0.05 two-tailed Student’s *t*-test, *n* = 6). In summary, photostimulation of D2R-MSNs in NAc activates local inhibitory circuits with postsynaptic neurons that are very similar to the D2R-MSNs but do not express D2R.

### Optogenetic stimulation of NAc D2R-MSNs in cocaine-induced behavioral sensitization

We next examined the behavioral consequences of *in vivo* photostimulation of NAc D2R-MSNs. Because photostimulation of D2R-MSNs in dorsal striatum decreases locomotor activity (Kravitz et al., 2010), we started by characterizing the effects of accumbens D2R-MSN activation on basal locomotor activity. For this purpose, D2R-Cre mice were injected with DIO-AAV-ChR2-EYFP virus bilaterally into the NAc (D2-Cre(+) NAc-ChR2). D2R-MSNs were then photostimulated with blue light (473 nm, 5 ms pulse duration, 20 Hz) delivered to the NAc via an optical fiber. Photostimuli were applied during four 3-min duration periods within the 50 min session when mice were kept in the locomotor activity recording chamber (Figure 4A). In parallel, as a control non-Cre WT littermate mice were similarly injected with virus and received similar blue light illumination. D2-Cre(+) NAc-ChR2 mice displayed a comparable or slightly elevated level of basal locomotor activity in comparison to the control D2R-Cre(−) NAc-ChR2 mice (Figures 4B,C). Photostimulation of D2R-MSNs in D2-Cre(+) NAc-ChR2 mice caused a significant decrease in the locomotor activity which recovered after the light stimulus stopped (Figure 4B). No such effects were observed in the control D2R-Cre(−) NAc-ChR2 mice (Figures 4B,C), indicating that the effects of photostimulation were caused by activation of ChR2, rather than possible non-specific effects such as heating of brain tissue. Therefore, our data indicated that photostimulation of D2R-MSNs in NAc elicited a decrease in locomotor activity.

These results established our ability to control the activity of D2R-MSNs within NAc *in vivo*. We next used this capability to examine the influence of D2R-MSN activity on behavioral sensitization to repeated administration of cocaine. Behavioral sensitization refers to the process that allows an initial exposure to psychostimulants, such as cocaine, to enhance the ability of subsequent drug exposures to stimulate locomotor activity. This process can be separated into initiation and expression phases: initiation describes the immediate neural events that induce behavioral sensitization (Vanderschuren and Kalivas, 2000; Sim et al., 2013), while expression is known to be a long-lasting form of behavioral plasticity that persists after drug withdrawal (Vanderschuren and Kalivas, 2000; Sim et al., 2013). We therefore examined cocaine-induced behavioral sensitization during repeated intraperitoneal (i.p.) injections of cocaine, while using optogenetics to control the activity of D2R-MSNs in NAc during each of these phases.

After habituation to saline injection over 3 days, mice were injected with cocaine (15 mg/kg) on 5 consecutive days and locomotor responses were recorded for 30 min after each injection (Figure 5A). Photostimuli were delivered during 30 min sessions before cocaine injection, interspersing 3 min periods of illumination with 5 min periods where the light was turned off (Figure 5A). Given that photostimulation of D2R-MSNs in NAc decreases basal locomotor activity (Figure 4), photostimuli were delivered immediately prior to administration of cocaine to avoid possible interference with behavioral responses to cocaine injection.

Both control D2-Cre(−) NAc-ChR2 mice and D2-Cre(+) NAc-ChR2 mice showed a marked increase in locomotor activity in response to the repeated cocaine injections (Figure 5B), indicating initiation of sensitization. Photostimulation of D2R-MSNs in NAc did not appear to affect the initiation of behavioral sensitization, because cocaine-induced behavioral sensitization was similar in D2-Cre(+) NAc-ChR2 mice and control D2-Cre(−) NAc-ChR2 mice.

After induction of behavioral sensitization by repeating such injections of cocaine (15 mg/kg) for 5 days, the drug was withdrawn for 14 days and the degree of expression of sensitization was examined by challenging the mice with a lower dose of cocaine (10 mg/kg). Expression of sensitization is a long-lasting form of behavioral plasticity that persists after drug withdrawal (Steketee and Kalivas, 2011; Sim et al., 2013). To examine the role of D2R-MSNs in expression of sensitization, NAc was photostimulated immediately prior to administration of cocaine (Figure 5A) and sensitization was measured as the amount of locomotor activity induced by the cocaine injection.

In both cocaine-pretreated groups of mice—D2-Cre(−) NAc-ChR2 mice (D2-Cre(−):: coc-coc) and D2-Cre(+) NAc-ChR2 (D2-Cre(+):: coc-coc)—robust expression of sensitization occurred (Figure 5C). The time course of cocaine-stimulated locomotion changes was also similar between the two groups (Figure 5C), with no significant difference observed between two groups. Taken together, these two photostimulation experiments indicate that activation of D2R-MSNs in the NAc does not affect initiation or expression of cocaine-induced behavioral sensitization.

### Photostimulation of NAc D2R-MSNs during drug withdrawal

Chronic stress during drug withdrawal after repeated cocaine exposure results in selective recruitment of a D2R-dependent adaptation mechanism that controls the stress-induced increase in cocaine-seeking and relapse behaviors in association with changes in synaptic plasticity in the NAc (Sim et al., 2013). This indicates that the mechanisms engaged by drug withdrawal are distinct from those involved in drug-indicted sensitization. We therefore next examined whether photostimulation of D2R-MSNs in NAc during cocaine withdrawal affects the expression of cocaine-induced behavioral sensitization.

After induction of behavioral sensitization by repeated injection of cocaine as above, D2-Cre(−) and D2-Cre(+) mice were subdivided into two groups for the 14 day withdrawal period: one group was subjected to daily blue-light stimulation of NAc for 1 h (3 min × 8 times), while the other group was not (Figure 6A). Repeated photostimulation of D2R-MSNs in NAc during cocaine withdrawal did not affect the expression of sensitization in D2-Cre(−):: coc-coc mice (Figure 6B). In contrast, in D2-Cre(+):: coc-coc mice, expression of sensitization was significantly attenuated by repeated photostimulation during drug withdrawal (Figure 6B), although the time course of the cocaine-induced stimulation of locomotion was unaffected (Figure 6C). Thus, photostimulation of D2R-MSNs of NAc during drug withdrawal reduced expression of cocaine-induced behavioral sensitization (cocaine × photo-stimulation interaction *F*_(1,18)_ = 11.08, *P* = 0.0037, Figure 6B). These data indicate that activation of D2R-NAc MSNs during the period of drug withdrawal influences cocaine-seeking and relapse behaviors.

## Discussion

Considerable evidence indicates that cocaine-induced behavioral sensitization is associated with enhanced dopaminergic transmission in the mesocorticolimbic system comprising the ventral tegmental area, prefrontal cortex and nucleus accumbens (NAc). In particular, the expression phase of behavioral sensitization is characterized by a persistent drug hyper-responsiveness after cessation of the drug, which is associated with a cascade of adaptation mechanisms (Kalivas and Duffy, 1990; Robinson and Berridge, 1993; Kalivas et al., 1998) that could contribute to compulsive drug craving (Robinson and Berridge, 1993; Kalivas et al., 1998; Steketee and Kalivas, 2011). It has been suggested that cocaine-induced alterations in molecular, cellular and behavioral plasticity within the NAc, in association with DA receptor signaling in MSNs, can regulate drug-mediated addictive behaviors (Lobo et al., 2010; Schmidt and Pierce, 2010; Ferguson et al., 2011; Pascoli et al., 2011; Bocklisch et al., 2013; Grueter et al., 2013).

Recent studies using genetically-engineered mice that conditionally express Cre recombinase have revealed roles for D1R-MSNs or D2R-MSNs in cocaine addictive behaviors. Optogenetic activation of D1R-MSNs of NAc after 6 days of repeated cocaine administration increases locomotor activity, while activation of D2R-MSNs reportedly has no effect (Lobo et al., 2010). These data suggest that repeated exposure to cocaine enhances the output of D1R-MSNs of the NAc. Inhibition of D1R-expressing MSNs with tetanus toxin (Hikida et al., 2010) diminishes cocaine-conditioned place preference (CPP), while no alterations in cocaine CPP were observed after abolishing synaptic transmission in D2R-MSNs (Hikida et al., 2010). Optogenetic activation of D1R-MSNs in dorsal striatum induces persistent reinforcement, whereas stimulating D2 receptor–expressing neurons induces transient punishment (Kravitz et al., 2012). A recent study has also reported that inhibition of D2R-MSNs via a chemicogenetic approach enhances the motivation to obtain cocaine, whereas optogenetic activation of D2R-MSNs suppresses cocaine self-administration (Bock et al., 2013). On the other hand, Bocklisch et al. (2013) reported that D1R-MSNs of the NAc project to the VTA, specifically to GABAergic neurons within the VTA, while D2R-MSNs do not project directly to the VTA. This circuit means that optogenetic activation of D1R-MSNs disinhibits DA neurons, which finally enhances cocaine-induced addictive behaviors (Bocklisch et al., 2013).

Despite the seemingly simple organization of these two populations of MSNs, the fact that MSNs receive multiple inputs and have different outputs from/to other brain areas, as well as forming local circuits between MSNs and other classes of interneurons, the resulting output of D1R-MSNs and D2R-MSNs can yield complex and different molecular, cellular and behavioral consequences.

Previously it has been shown that D2R contributes to synaptic modifications induced during drug withdrawal and these potentiate the relapse to cocaine seeking, without affecting initial drug acquisition or drug-seeking (Sim et al., 2013). Our present data indicate that photostimulation of D2R-MSNs in NAc elicits a decrease in basal locomotor activity. Lobo et al. (2010) detected no change in locomotion when either MSN subtype was activated, but they only examined total locomotor activity rather than examining immediate responses of basal locomotor activity to photostimulation. Kravitz et al. (2010) also found that optogenetic activation of D2R-MSNs in dorsal striatum also decreases locomotor activity. Thus, our data are the first to demonstrate that basal locomotor activity is inhibited by photostimulation of D2R-MSNs of NAc and the first to systematically examine the time course of basal locomotor activity during photostimulation of these neurons.

In the present study, we observed that optogenetic activation of D2R-MSNs in NAc did not affect the initiation or expression of behavioral sensitization. However, photostimulation of D2R-MSNs during the drug withdrawal period blunted the expression of cocaine-induced sensitization. Therefore, our data indicate that D2R-MSNs are recruiting some signal specifically during the withdrawal period that goes on to alter gene expression or other forms of signaling and thereby trigger changes in synaptic plasticity, leading to alterations in cocaine-induced behavioral sensitization. How these MSNs employ cell-type specific adaptations that can produce their distinct consequences in addiction-related behaviors is not known. Grueter et al. (2013) suggested that ΔFosB in NAc differentially modulates synaptic properties and reward-related behaviors in a cell type- and subregion-specific fashion. Recently, Chandra et al. (2013) reported that repeated ChR2 activation of D1R-MSNs but not D2R-MSNs caused a down-regulation of Tiam1 gene, a protein involved in the rearrangement of the actin cytoskeleton, similar to the effects of cocaine. Therefore, to understand the mechanisms that yield lasting effects of drug-induced behaviors it will be important to delineate the cell-selective induction of molecular events in these MSNs that control synaptic adaptation to repetitive drug exposure.

In association with repetitive drug exposure, withdrawal has been suggested to play an important role because some changes appear only several weeks after the final exposure to cocaine. This suggests that abstinence is an important mediator in development of plasticity (Robinson and Berridge, 2003; Boudreau and Wolf, 2005; Boudreau et al., 2007; Kourrich et al., 2007). These observations raise the possibility that withdrawal itself might be a trigger for the changes in the NAc that are under the control of D2R-dependent signaling. Our result showing that activation of D2R-MSNs in NAc during drug withdrawal affect cocaine-induced behavioral sensitization provides compelling support for this idea.

It has previously been shown that repeated exposure to stress during drug withdrawal suppresses expression of cocaine-induced behavioral sensitization as well as cocaine-seeking and relapse behaviors in D2R KO mice (Sim et al., 2013). It is therefore interesting that photostimulation of D2R-MSNs during drug withdrawal also attenuates expression of sensitization. Stress-induced synaptic plasticity at glutamategic synapses is altered in the NAc of D2R KO mice (Sim et al., 2013). Although it is not yet known whether photostimulation of D2R MSNs or chronic stress during the withdrawal period elicits similar alterations in synaptic plasticity, our present findings support the hypothesis that D2R-MSNs of NAc play a key role in withdrawal-induced plasticity and may contribute to relapse after cessation of drug abuse. Further investigation will be required to find out the functional neural circuits in which D2R MSNs participate during drug withdrawal and to analyze and compare the consequences of D2R-MSNs photostimulation and chronic stress on synaptic plasticity in this particular circuit.

Another possible role for D2R-expressing MSNs could be to inhibit the output of D1R-MSNs from NAc. Previous research indicates that although MSNs project long axons to remote targets, extensive overlap occurs between axon collaterals and the dendritic trees of adjacent spiny projection neurons (Grofová, 1975; Preston et al., 1980; Wilson and Groves, 1980). This could indicate possible local synaptic connectivity for MSN within the NAc. Intracellular recordings from pairs of spiny projection neurons have identified functional inhibitory connections between MSNs in rat striatum (Czubayko and Plenz, 2002; Tunstall et al., 2002; Koos et al., 2004; Gustafson et al., 2006). It has been also reported that the synapses formed by recurrent collateral axons of MSNs in striatum are not random, D2R-MSNs make synaptic connections both with other D2R-MSNs and with D1R-MSNs, whereas D1R-MSNs almost exclusively form synaptic connections with other D1R-MSNs (Taverna et al., 2008). Although the GABAergic interconnection by local recurrent axonal collaterals between accumbal MSNs have also been reported (Taverna et al., 2004), it is still not clear yet whether D2R-MSNs randomly form local microcircuits or they contribute to microcircuits in NA with preferential connection as they do in striatum. Our data suggest that D2R-MSNs in NAc expressing ChR2 make synaptic connections with neighboring MSNs that express D1R, and that D2R-MSNs then exert an inhibitory contact to D1-MSNs to modulate the D1R-mediated promotion of addictive behaviors.

In conclusion, we have shown that optogenetic activation of NAc D2R-MSNs alters withdrawal-induced plasticity occurring during cocaine addiction. Given that activity of D2R-dependent signaling during the withdrawal period appears to be a key regulator of the expression of cocaine-induced behavioral sensitization, we propose that D2R-MSNs are an important mediator of long-lasting adaptation for drug-seeking and relapse. The identification of molecular substrates of D2R-dependent signaling, together with identification of specific circuit of NAc D2R-MSNs employed under repetitive drug exposure, should provide novel targets for therapeutic intervention in drug relapse.

## Conflict of interest statement

The authors declare that the research was conducted in the absence of any commercial or financial relationships that could be construed as a potential conflict of interest.

## References

[B1] Baik J. H. (2013). Dopamine signaling in reward-related behaviors. Front. Neural Circuits 7:152 10.3389/fncir.2013.00152 24130517 PMC3795306

[B2] Baik J. H. Picetti R. Saiardi A. Thiriet G. Dierich A. Depaulis A. (1995). Parkinsonian-like locomotor impairment in mice lacking dopamine D2 receptors. Nature 377, 424–428 10.1038/377424a0 7566118

[B3] Berridge K. C. (2007). The debate over dopamine’s role in reward: the case for incentive salience. Psychopharmacology (Berl) 191, 391–431 10.1007/s00213-006-0578-x 17072591

[B4] Bock R. Shin J. H. Kaplan A. R. Dobi A. Markey E. Kramer P. F. (2013). Strenghtening the accumbal indirect pathway promotes resilience to compulsive cocaine use. Nat. Neurosci. 16, 632–638 10.1038/nn.3369 23542690 PMC3637872

[B5] Bocklisch C. Pascoli V. Wong J. C. House D. R. Yvon C. de Roo M. (2013). Cocaine disinhibits dopamine neurons by potentiation of GABA transmission in the ventral tegmental area. Science 341, 1521–1525 10.1126/science.1237059 24072923

[B6] Boudreau A. C. Reimers J. M. Milovanovic M. Wolf M. E. (2007). Cell surface AMPA receptors in the rat nucleus accumbens increase during cocaine withdrawal but internalize after cocaine challenge in association with altered activation of mitogen-activated protein kinases. J. Neurosci. 27, 10621–10635 10.1523/jneurosci.2163-07.2007 17898233 PMC2856315

[B7] Boudreau A. C. Wolf M. E. (2005). Behavioral sensitization to cocaine is associated with increased AMPA receptor surface expression in the nucleus accumbens. J. Neurosci. 25, 9144–9151 10.1523/jneurosci.2252-05.2005 16207873 PMC6725751

[B8] Chandra R. Lenz J. D. Gancarz A. M. Chaudhury D. Schroeder G. L. Han M. H. (2013). Optogenetic inhibition of D1R containing nucleus accumbens neurons alters cocaine-mediated regulation of Tiam1. Front. Mol. Neurosci. 6:13 10.3389/fnmol.2013.00013 23745104 PMC3662885

[B9] Chang H. T. Kitai S. T. (1985). Projection neurons of the nucleus accumbens: an intracellular labeling study. Brain Res. 347, 112–116 10.1016/0006-8993(85)90894-7 2996712

[B10] Chausmer A. L. Elmer G. I. Rubinstein M. Low M. J. Grandy D. K. Katz J. L. (2002). Cocaine-induced locomotor activity and cocaine discrimination in dopamine D2 receptor mutant mice. Psychopharmacology (Berl) 163, 54–61 10.1007/s00213-002-1142-y 12185400

[B11] Chevalier G. Deniau J. M. (1990). Disinhibition as a basic process in the expression of striatal functions. Trends Neurosci. 13, 277–280 10.1016/0166-2236(90)90109-n 1695403

[B46] Czubayko U. Plenz D. (2002). Fast synaptic transmission between striatal spiny projection neurons. Proc. Natl. Acad. Sci. U S A 99, 15764–15769 10.1073/pnas.242428599 12438690 PMC137790

[B12] Ferguson S. M. Eskenazi D. Ishikawa M. Wanat M. J. Phillips P. E. Dong Y. (2011). Transient neuronal inhibition reveals opposing roles of indirect and direct pathways in sensitization. Nat. Neurosci. 14, 22–24 10.1038/nn.2703 21131952 PMC3058296

[B14] Goto Y. Grace A. A. (2005). Dopaminergic modulation of limbic and cortical drive of nucleus accumbens in goal-directed behavior. Nat. Neurosci. 8, 805–812 10.1038/nn1471 15908948

[B15] Grofová I. (1975). The identification of striatal and pallidal neurons projecting to substantia nigra. An experimental study by means of retrograde axonal transport of horseradish peroxidase. Brain Res. 91, 286–291 10.1016/0006-8993(75)90550-8 51667

[B16] Grueter B. A. Robison A. J. Neve R. L. Nestler E. J. Malenka R. C. (2013). ΔFosB differentially modulates nucleus accumbens direct and indirect pathway function. Proc. Natl. Acad. Sci. U S A 110, 1923–1928 10.1073/pnas.1221742110 23319622 PMC3562792

[B47] Gustafson N. Gireesh-Dharmaraj E. Czubayko U. Blackwell K. T. Plenz D. (2006). A comparative voltage and current-clamp analysis of feedback and feedforward synaptic transmission in the striatal microcircuit in vitro. J. Neurophysiol. 95, 737–752 10.1152/jn.00802.2005 16236782

[B17] Hikida T. Kimura K. Wada N. Funabiki K. Nakanishi S. (2010). Distinct roles of synaptic transmission in direct and indirect striatal pathways to reward and aversive behavior. Neuron 66, 896–907 10.1016/j.neuron.2010.05.011 20620875

[B18] Kalivas P. W. Duffy P. (1990). Effect of acute and daily cocaine treatment on extracellular dopamine in the nucleus accumbens. Synapse 5, 48–58 10.1002/syn.890050104 2300906

[B19] Kalivas P. W. Pierce R. C. Cornish J. Sorg B. A. (1998). A role for sensitization in craving and relapse in cocaine addiction. J. Psychopharmacol. 12, 49–53 10.1177/026988119801200107 9584968

[B48] Koos T. Tepper J. M. Wilson C. J. (2004). Comparison of IPSCs evoked by spiny and fast-spiking neurons in the neostriatum. J. Neurosci. 24, 7916–7922 10.1523/jneurosci.2163-04.2004 15356204 PMC6729926

[B20] Kourrich S. Rothwell P. E. Klug J. R. Thomas M. J. (2007). Cocaine experience controls bidirectional synaptic plasticity in the nucleus accumbens. J. Neurosci. 27, 7921–7928 10.1523/jneurosci.1859-07.2007 17652583 PMC6672735

[B21] Kravitz A. V. Freeze B. S. Parker P. R. Kay K. Thwin M. T. Deisseroth K. (2010). Regulation of parkinsonian motor behaviours by optogenetic control of basal ganglia circuitry. Nature 466, 622–626 10.1038/nature09159 20613723 PMC3552484

[B22] Kravitz A. V. Tye L. D. Kreitzer A. C. (2012). Distinct roles for direct and indirect pathway striatal neurons in reinforcement. Nat. Neurosci. 15, 816–818 10.1038/nn.3100 22544310 PMC3410042

[B23] Kreitzer A. C. Malenka R. C. (2008). Striatal plasticity and basal ganglia circuit function. Nature 60, 543–554 10.1016/j.neuron.2008.11.005 19038213 PMC2724179

[B24] Le Moine C. Bloch B. (1996). Expression of the D3 dopamine receptor in peptidergic neurons of the nucleus accumbens: comparison with the D1 and D2 dopamine receptors. Neuroscience 73, 131–143 10.1016/0306-4522(96)00029-2 8783237

[B26] Lobo M. K. Covington H. E. 3rd Chaudhury D. Friedman A. K. Sun H. Damez-Werno D. (2010). Cell type-specific loss of BDNF signaling mimics optogenetic control of cocaine reward. Science 330, 385–390 10.1126/science.1188472 20947769 PMC3011229

[B27] Lobo M. K. Nestler E. J. (2011). The striatal balancing act in drug addiction: distinct roles of direct and indirect pathway medium spiny neurons. Front. Neuroanat. 5:41 10.3389/fnana.2011.00041 21811439 PMC3140647

[B28] Lüscher C. Malenka R. C. (2011). Drug-evoked synaptic plasticity in addiction: from molecular changes to circuit remodeling. Neuron 69, 650–663 10.1016/j.neuron.2011.01.017 21338877 PMC4046255

[B29] O’Donnell P. Grace A. A. (1993). Physiological and morphological properties of accumbens core and shell neurons recorded in vitro. Synapse 13, 135–160 10.1002/syn.890130206 8446922

[B30] Pascoli V. Turiault M. Lüscher C. (2011). Reversal of cocaine-evoked synaptic potentiation resets drug-induced adaptive behaviour. Nature 481, 71–75 10.1038/nature10709 22158102

[B31] Preston R. J. Bishop G. A. Kitai S. T. (1980). Medium spiny neuron projection from the rat neostriatum: an intracellular horseradish peroxidase study. Brain Res. 183, 253–263 10.1016/0006-8993(80)90462-x 7353139

[B32] Robinson T. E. Berridge K. C. (1993). The neural basis of drug craving: an incentive-sensitization theory of addiction. Brain Res. Brain Res. Rev. 18, 247–291 10.1016/0165-0173(93)90013-p 8401595

[B33] Robinson T. E. Berridge K. C. (2003). Addiction. Annu. Rev. Psychol. 54, 25–53 10.1146/annurev.psych.54.101601.145237 12185211

[B34] Schmidt H. D. Pierce R. C. (2010). Cocaine-induced neuroadaptations in glutamate transmission: potential therapeutic targets for craving and addiction. Ann. N Y Acad. Sci. 1187, 35–75 10.1111/j.1749-6632.2009.05144.x 20201846 PMC5413205

[B35] Sesack S. R. Grace A. A. (2010). Cortico-basal ganglia reward network: microcircuitry. Neuropsychopharmacology 35, 27–47 10.1038/npp.2009.93 19675534 PMC2879005

[B36] Sim H. R. Choi T. Y. Lee H. J. Kang E. Y. Yoon S. Han P. L. (2013). Role of dopamine D2 receptors in plasticity of stress-induced addictive behaviours. Nat. Commun. 4:1579 10.1038/ncomms2598 23481387

[B37] Smith R. J. Lobo M. K. Spencer S. Kalivas P. W. (2013). Cocaine-induced adaptations in D1 and D2 accumbens projection neurons (a dichotomy not necessarily synonymous with direct and indirect pathways). Curr. Opin. Neurobiol. 23, 546–552 10.1016/j.conb.2013.01.026 23428656 PMC3681928

[B38] Steketee J. D. Kalivas P. W. (2011). Drug wanting: behavioral sensitization and relapse to drug-seeking behavior. Pharmacol. Rev. 63, 348–365 10.1124/pr.109.001933 21490129 PMC3082449

[B39] Taverna S. Ilijic E. Surmeier D. J. (2008). Recurrent collateral connections of striatal medium spiny neurons are disrupted in models of Parkinson’s disease. J. Neurosci. 28, 5504–5512 10.1523/JNEUROSCI.5493-07.2008 18495884 PMC3235738

[B40] Taverna S. van Dongen Y. C. Groenewegen H. J. Pennartz C. M. (2004). Direct physiological evidence for synaptic connectivity between medium-sized spiny neurons in rat nucleus accumbens in situ. J. Neurophysiol. 91, 1111–1121 10.1152/jn.00892.2003 14573550

[B41] Thomas M. J. Kalivas P. W. Shaham Y. (2008). Neuroplasticity in the mesolimbic dopamine system and cocaine addiction. Br. J. Pharmacol. 154, 327–342 10.1038/bjp.2008.77 18345022 PMC2442442

[B42] Tunstall M. J. Oorschot D. E. Kean A. Wickens J. R. (2002). Inhibitory interactions between spiny projection neurons in the rat striatum. J. Neurophysiol. 88, 1263–1269 10.1152/jn.00886.2001 12205147

[B43] Vanderschuren L. J. Kalivas P. W. (2000). Alterations to dopaminergic and glutamatergic transmission in the induction and expression of behavioral sensitization: a critical review of preclinical studies. Psychopharmacology (Berl) 151, 99–120 10.1007/s002130000493 10972458

[B44] Wilson C. J. Groves P. M. (1980). Fine structure and synaptic connection of the common spiny neuron of the rat neostriatum: a study employing intracellular injection of horseradish peroxidase. J. Comp. Neurol. 194, 599–615 10.1002/cne.901940308 7451684

[B45] Wise R. A. (2004). Dopamine, learning and motivation. Nat. Rev. Neurosci. 5, 483–494 10.1038/nrn1406 15152198

